# Voltammetric sensor based on long alkyl chain tetraalkylammonium ionic liquids comprising ascorbate anion for determination of nitrite

**DOI:** 10.1007/s00604-021-04713-4

**Published:** 2021-01-27

**Authors:** Tomasz Rębiś, Michał Niemczak, Patrycja Płócienniczak, Juliusz Pernak, Grzegorz Milczarek

**Affiliations:** 1 Institute of Chemistry and Technical Electrochemistry, Poznan University of Technology, Berdychowo 4, 60-965 Poznan, Poland; 2 Department of Chemical Technology, Poznan University of Technology, ul. Berdychowo 4, 60-965 Poznan, Poland

**Keywords:** Long alkyl chain tetraalkylammonium ionic liquid, Nitrite, Voltammetry, Curing salt

## Abstract

An electrochemical sensor was fabricated utilizing ionic liquids possessing cations with long alkyl chains such as trimethyl octadecylammonium and behenyl trimethylammonium and ascorbate anion. The ionic liquids were drop-coated onto the electrode. Thin modifying layers were prepared. Cyclic voltammetric investigations revealed electrostatic interactions between the electrochemical probes and the modified surface, proving that a positive charge was established at the film surface. Hence, negatively charged species such as nitrite can be pre-concentrated on the surface of presented modified electrodes. The fabricated electrodes have been used as a voltammetric sensor for nitrite. Due to the electrostatic accumulation properties of long alkyl cation, the assay exhibits a remarkable improvement in the voltammetric response toward nitrite oxidation. The influence of pH on the electrode response was thoroughly investigated, and the mechanism of the electrode was established. The developed sensor showed a linear electrochemical response in the range 1.0–50 μM with a detection limit of 0.1 μM. The electrode revealed good storage stability, reproducibility, and anti-interference ability. The determination of nitrite performed in curing salts brought satisfactory results.

**Supplementary Information:**

The online version contains supplementary material available at 10.1007/s00604-021-04713-4.

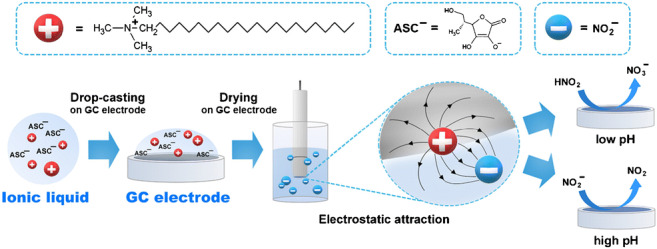

## Introduction

Nitrite is an ion commonly found in food, soil, water, and different physiological systems. Great amounts of nitrite compounds are used in food technology, especially as preservatives. Nitrite is used as an additive in cured products to obtain the characteristic red color and desirable flavor [1, 2]. Nitrite is potentially toxic and irritant, which is why it is considered a pollutant and a cause of deterioration of human health [3]. The excess level of nitrite in the blood is hazardous. Different studies have proved that nitrite can undergo a reaction with secondary amines to produce mostly carcinogenic nitrosamines [4]. Therefore, reliable and accurate determination of nitrate substances in the environment is very important [5–7].

To date, numerous methods including chromatography, chemiluminescence, spectrophotometry, and electrochemical sensors have been developed to detect nitrite [3, 4, 8]. Low-cost instrumentation, fast response, and simplicity of use made the electrochemical approach a useful tool for the determination of nitrite [6, 9–12]. However, the voltammetric determination of nitrite encounters difficulties associated with slow electron transfer kinetics, which in turn requires the large overpotentials to be involved. Therefore, to obtain better sensitivity and lower overpotential for the oxidation of various compounds, it is necessary to modify the electrode surface with electroactive materials [5, 7, 13–15].

Owing to the need for a high performance of the electrochemical nitrite sensor, the surface modifications of the electrode are usually applied to boost their sensitivity and to enhance selective interactions between the sensing layer and analytes. In recent years, various chemically modified electrodes have been proposed for the determination of nitrites. For instance, poly(3,4-ethylenedioxythiophene) (PEDOT) hollow microflower was applied [16]. Platinum nanoparticles embedded on polypyrrole matrix were also reported as a sensor for nitrite detection [17]. Various redox mediators such as metalled phthalocyanines or porphyrazines were used to build nitrite sensitive modified electrodes. In particular, macrocycles bearing transition metal ions such as Co, Fe, and Ni in their cores and involving reversible redox transitions are considered to be suitable for nitrite determination [3, 7, 18–20]. Recently, composites consisting of graphene nanoparticles and precious metal have also been given attention [6, 9]. Furthermore, papers describing modified electrodes based on various nickel materials enabling electrocatalytic detection of nitrite have been published [7, 13, 19, 21].

Over the last few years, the use of ionic liquids (ILs) for electrochemical sensing has gained momentum. ILs have demonstrated many attractive physicochemical properties such as good ionic conductivity, high chemical and thermal stability, and wide electrochemical window. It has been presented that ILs can be effectively used as the high-performance modifiers for the development of modified electrodes [22–24]. Recent results suggest that the ionic liquid/graphene modified electrode can be utilized for simultaneous electrochemical sensing of thallium, lead, and mercury [25]. Moreover, the electrochemical oxidation of NADH on a glassy carbon electrode modified by IL (1-butyl-3-methylimidazolium tetrafluoroborate, BMIM·BF_4_) and multi-walled carbon nanotubes with a polymeric matrix of chitosan was also reported [26]. The IL applied as active material in the preparation of modified electrode for nitrite oxidation has also been evaluated. For instance, Wang et al. presented Prussian blue/1-butyl-3-methylimidazolium tetrafluoroborate–graphite felt electrodes for the efficient electrocatalytic determination of nitrite [27]. Moreover, the electrode with ionic liquid n-octylpyridinum hexafluorophosphate (OPFP) and single-walled carbon nanotube has also been proposed [28].

In this work, the electrodes were modified with thin layers of ILs containing cation substituted with long alkyl chains such as trimethyl octadecylammonium (C_18_TMA-ASC) and behenyl trimethylammonium (C_22_TMA-ASC). As a result of the proposed modification, the oxidation current of nitrite increases significantly when compared to the currents recorded on an unmodified glassy carbon (GC) electrode. The increase of recorded signals is caused by electrostatic interactions between the nitrate anion (NO_2_^−^) and the cationic form of ionic liquids covering the electrode surface. The practical application of the presented sensor was evaluated in commercially available curing salt samples under validated conditions. To the best of the authors’ knowledge, there is yet no report regarding the application of long alkyl ammonium ILs in the development of electrochemical sensors.

## Experimental part

### Materials and reagents

**Scheme 1:**
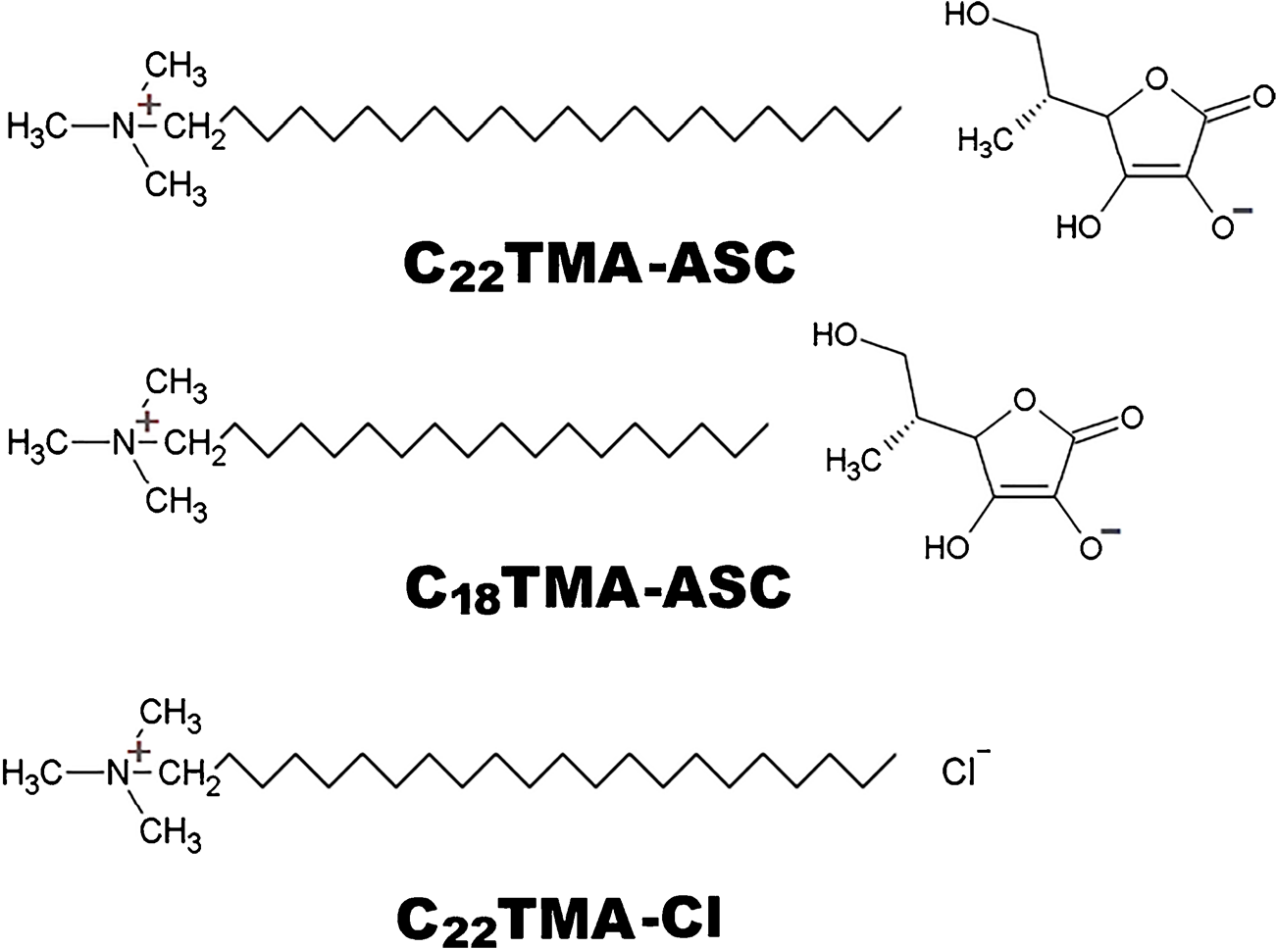
Chemical structures of the studied ILs

ILs comprising of ascorbate anion and cation substituted with long alkyl chain such as trimethyl octadecylammonium (C_18_TMA-ASC) and behenyl trimethylammonium (C_22_TMA-ASC) were synthesized according to a previously published procedure [29]. Both ILs were synthesized under mild conditions using ion-exchange resin, which allows products deprived of impurities in the form of chlorides to be obtained. Firstly, the appropriate quaternary ammonium chloride was dissolved in ethanol and then mixed with the anionic resin Dowex Monosphere 550A in the form of an ethanolic suspension. The mixture was stirred for 1 h at 25 °C, and thereby, the chloride anions were replaced with hydroxide ions. Subsequently, the intermediates (organic ammonium hydroxides) were neutralized with ascorbic acid in a 1:1 stoichiometric ratio using an Easy-Max reactor. Then, after evaporation of the solvent, the obtained products were dried under a vacuum at 25 °C for 24 h and stored over P_4_O_10_ at −20 °C. Structures of ILs are presented in Scheme 1.

Sodium nitrite (NaNO_2_) and dimethylformamide (DMF) were purchased from Sigma-Aldrich. Perchloric acid, monosodium, and disodium phosphates for the preparation of phosphate buffer (PB) were provided by Avantor POCH (Gliwice, Poland). All these compounds were of reagent grade and were used without further purification.

Britton-Robinson buffers were prepared by adding the desired volume of 0.2 M NaOH to the mixture of 0.04 M H_3_BO_3_, 0.04 M H_3_PO_4_, and 0.04 M CH_3_COOH. All solutions were prepared using deionized water.

The commercial curing salts (3 samples) were purchased from a local supermarket. To fit within a proper nitrite concentration, the accurate mass of the salt sample was weighed, dissolved with electrolyte, and diluted to 25.0 mL in a calibrated flask. The nitrite determination was carried out by differential pulse voltammetric measurements (DPVs) using the standard addition method. The first voltammetric signal was due to the addition of the curing salt solution. Next, the aliquots (5 μM) of nitrites were added to create a standard addition plot. The concentration of nitrites in curing salt is the reading of the unknown.

### Electrochemical methods

Electrochemical measurements were performed using a μ-Autolab III (ECO Chemie, The Netherlands) potentiostat/galvanostat. A typical three-electrode configuration was used with Ag/AgCl/3M KCl as a reference electrode, a Pt wire as a counter electrode, and a glassy carbon disk (GC, d = 3 mm, BasiINC, USA) as working electrode. Before the electrochemical experiments, a GC electrode had been polished with aqueous 50 nm Al_2_O_3_ slurry (Buehler) on a polishing cloth, followed by subsequent washing in an ultrasonic bath with acetone for 10 min in order to remove any trace impurities.

### Fabrication of GC/C_18_TMA-ASC, GC/C_22_TMA-Cl, and GC/C_22_TMA-ASC modified electrodes

In order to modify the surface of the GC electrode, 10 mM solutions of studied ILs were dissolved in DMF in the first step. Next, the thin layers were deposited onto the GC by dropping 1, or 3 μL of each IL. The surfaces were subsequently dried at room temperature for ca. 1 h. The electrodes prepared this way were ready to use in electrochemical measurements. As a pretreatment, the electrodes were immersed in pure phosphate buffer (PB, pH 7.4) and scanned from − 0.2 to 0.6 V (20 cycles) to obtain a stable cyclic voltammogram (CV). Final electrodes were denoted as GC/C_18_TMA-ASC, GC/C_22_TMA-Cl, and GC/C_22_TMA-ASC.

## Results and discussion

### Electrochemical characterization of GC/C_18_TMA-ASC, GC/C_22_TMA-Cl, and GC/C_22_TMA-ASC electrodes

**Fig. 1:**
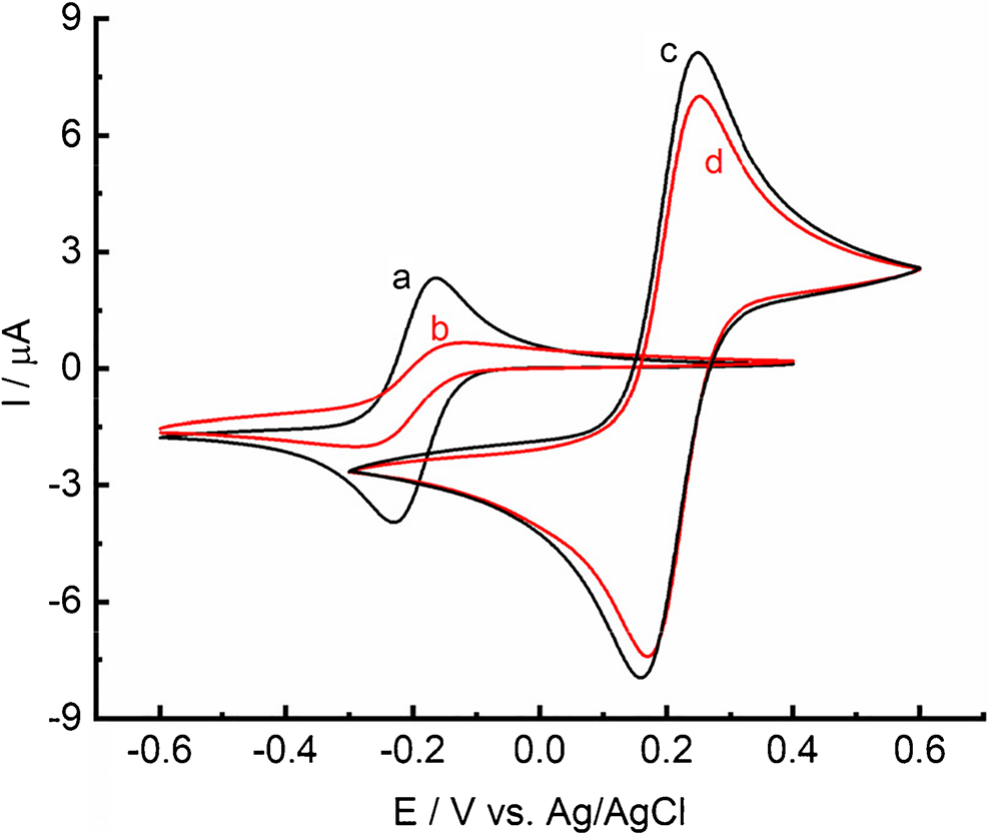
CVs in the presence of 1 mM of Ru(NH_3_)_6_^3+^ recorded at bare GC (a) and GC/C_22_TMA-ASC (b) as well as CVs in the presence of 1 mM Fe(CN)_6_^3−/4−^ recorded at bare GC (c) and GC/C_22_TMA-ASC (d). The supporting electrolyte was PB solution (pH 7.4). Scan rate 10 mV s^−1^

In Fig. 1, cyclic voltammograms for GC/C_22_TMA-ASC electrode recorded in PB buffer in the presence of redox markers Ru(NH_3_)_6_^3+^ and Fe(CN)_6_^3−/4−^ are shown. According to the results, a decrease in current peaks is observed in the presence of both positively charged Ru(NH_3_)_6_^3+^ and negatively charged Fe(CN)_6_^3−/4−^. The observed behavior is particularly pronounced in the case of Ru(NH_3_)_6_^3+^. These results indicate a partial blocking of the electrode surface by a thin layer of C_22_TMA-ASC modifier [30, 31]. Since the modifying IL has a long hydrophobic chain, which is a cation, electrostatic interactions between the thin layer of the modifier and ionic redox compounds can be expected. In the case of Ru(NH_3_)_6_^3+^, a significant increase in peak separation from 63 to 172 mV (Fig. 1, curves a and b) is observed at the GC/C_22_TMA-ASC electrode, which suggests coulombic repulsion of the positively charged electrode surface with Ru(NH_3_)_6_^3+^ cation.

The opposite effect is observed when an anionic marker (Fe(CN)_6_^3−/4−^) is applied. A slight decrease in the separation of peaks from 80 to 75 mV is observed, which may indicate adsorption of Fe(CN)_6_^3−/4−^ on the electrode surface (Fig. 1, curves c and d). This effect can be ascribed to the electrostatic attraction of the Fe(CN)_6_^3−/4−^ anion by the behenyl trimethylammonium cation. The CVs recorded for both Fe(CN)_6_^3−/4−^ and Ru(NH_3_)_6_^3+^ redox markers at GC/C_18_TMA-ASC and GC/C_22_TMA-Cl electrodes are displayed in Fig. S1 and Fig. S2, respectively. As it can be seen, similar results to those at GC/C_22_TMA-ASC are observed. According to the given data, the influence of electrostatic interactions is the weakest for the GC/C_18_TMA-ASC electrode. Table 1 summarizes the peak separation for Fe(CN)_6_^3−/4−^ and Ru(NH_3_)_6_^3+^ redox markers recorded for the GC/C_18_TMA-ASC, GC/C_22_TMA-Cl, and GC/C_22_TMA-ASC electrodes tested.

**Table 1:** Values of ΔE and I_peak_ for selected redox markers recorded at studied modified electrodes

Redox marker		GC	GC/C_22_TMA-ASC	GC/C_18_TMA-ASC	GC/C_22_TMA-Cl
Fe(CN)_6_^3−/4-^	ΔE/mV	80	75	80	79
	I_peak_/μA	10.4	9.5	9.7	10.0
Ru(NH_3_)_6_^3+^	ΔE/mV	63	172	120	165
	I_peak_/μA	4.1	1.4	3.8	2.1

### Voltammetric response of nitrite at the GC/C_18_TMA-ASC, GC/C_22_TMA-Cl, and GC/C_22_TMA-ASC electrodes

**Fig. 2:**
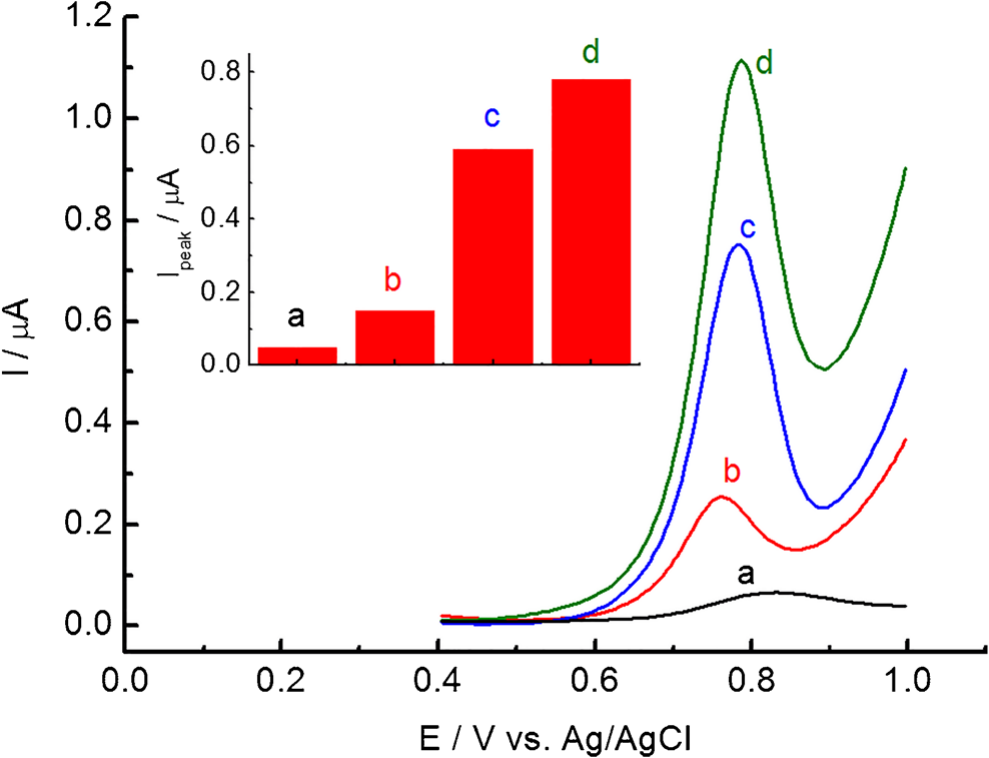
DPV response for bare GC (a), GC/C_18_TMA-ASC (b), GC/C_22_TMA-Cl (c), and GC/C_22_TMA-ASC (d) in the presence of 10 μM NaNO_2_. The inset presents corresponding peak currents (I_peak_) recorded in the presence of 10 μM NaNO_2_ at bare GC (a), GC/C_18_TMA-ASC (b), GC/C_22_TMA-Cl (c), and GC/C_22_TMA-ASC (d)

The DPV responses for the oxidation of 10 μM NaNO_2_ at bare GC, GC/C_18_TMA-ASC, GC/C_22_TMA-Cl, and GC/C_22_TMA-ASC were studied, and the results are presented in Fig. 2. As shown, all the modified electrodes reveal a well-defined and sharp peak that appears in the presence of 10 μM NaNO_2_. The voltammetric signals are significantly higher as compared to the bare GC electrode. Moreover, the peaks for NaNO_2_ oxidation at the modified electrodes were recorded at lower potentials as compared to the bare GC. Such behavior suggests the adsorption/pre-concentration ability of the modified layers toward NaNO_2_. Poor current response in the case of GC is related to sluggish kinetics for nitrite oxidation as previously reported for this material [7, 20]. Among the electrodes studied here, the GC/C_22_TMA-ASC showed the highest voltammetric peak. These results indicate much higher sensitivity of the GC/C_22_TMA-ASC electrode than that of a bare GC. The improved electrochemical response recorded at GC/C_22_TMA-ASC for nitrite can be assigned to the positively charged long alkyl behenyl trimethylammonium groups on the surface of the electrode. The electrostatic accumulation of nitrite at the electrode surface can promote its surface concentration and, consequently, boost the oxidation signal of NaNO_2_.

To verify the influence of electrode thickness (mass loading) on the redox peak of nitrate oxidation, a series of DPV measurements were conducted in the presence of 5 μM NaNO_2_. DPVs were recorded at the GC/C_22_TMA-ASC electrodes containing different modifier masses in the range from 5.5 to 16.5 μg. As shown in Fig. S3, the increase in the mass of the C_22_TMA-ASC from 5.5 to 11.0 μg causes the over twofold increase in the current signal, which indicates the enhanced accumulation effect of the electrode. However, the decrease in the peak current is observed when the electrode mass rises to 16.5 μg. It is probably related to the diffusion limitations that may appear at thick layers of the modifier [32]. Thus, 11.0 μg of C_22_TMA-ASC was chosen as the optimal mass and the electrode prepared in this way was used in further experimental studies.

### Effect of pH, peak current, and scan rate at the GC/C_22_TMA-ASC electrode

**Fig. 3:**
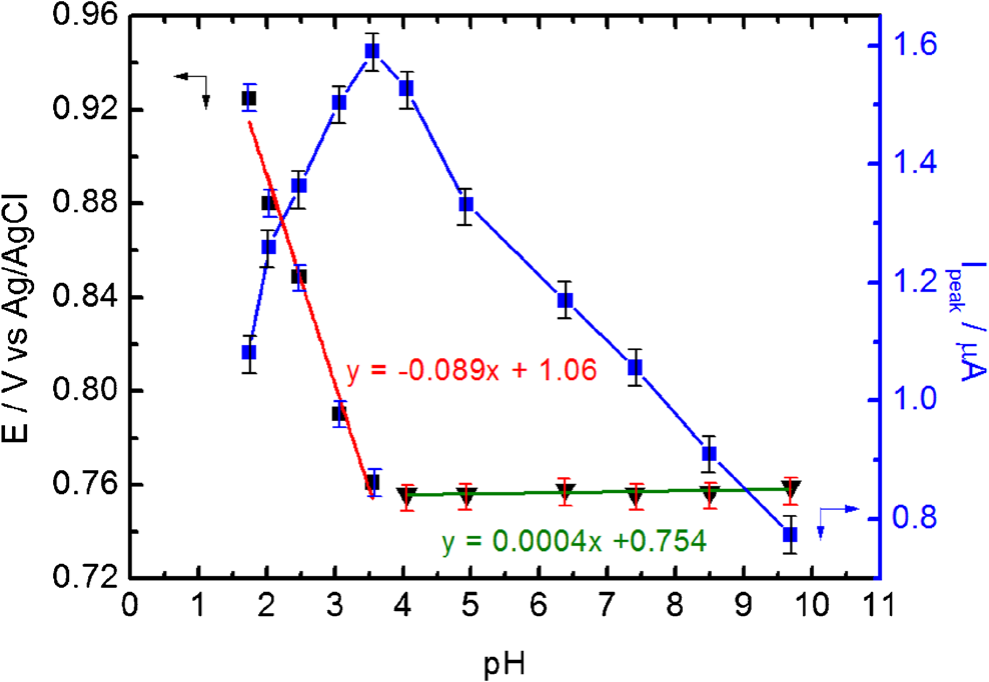
The influence of pH on the peak potential and the peak current of NaNO_2_ oxidation (c = 10 μM) at the GC/C_22_TMA-ASC electrode (*n* = 3)

We also carried out the studies on the influence of pH on two variables such as peak current and peak potential, and the results are presented in Fig. 3. The obtained plots show clearly that at low pH (from 1.0 to 3.5) we can observe a significant increase in peak current with parallelly decreasing peak potential. At higher pH values (between 3.5 and 9.6), the peak potential remains almost constant; however, the peak current starts to decrease significantly. The shift of the peak potential as a function of pH suggests that protons participate in redox reaction in low pH. However, the electrode mechanism in high pH does not involve protons.

HNO_2_ is a week acid with pK_a_ equal to 3.3. Hence, at pH below 3.3 nitrous acid exists in protonated form according to chemical equilibrium (Eq. 1):

1 $$ {\mathrm{H}\mathrm{NO}}_2+{\mathrm{H}}_2\mathrm{O}\to {{\mathrm{NO}}_2}^{-}+{\mathrm{H}}_3{\mathrm{O}}^{+} $$

The obtained results show that the protonation degree of nitrite strongly affects the nitrite oxidation. At low pH, the molecular form of the weak acid is preferred, so that the electrostatic attraction between the cationic layer of the modifier and the anionic analyte can be limited. The higher the pH, the higher the deprotonation; thus, electrostatic attraction between the electrode surface and analyte increases. As a result, the oxidation peak remarkably increased when the pH went up and reached the maximum value of 3.5. Similar behavior was previously observed for a multiwalled carbon nanotube paste electrode modified with chitosan-functionalized silver nanoparticles, where charged amino groups were responsible for the electrostatic attraction of nitrites [33, 34]. On the other hand, it is well-known that nitrous acid is not stable in a strongly acidic electrolyte and can undergo the following disproportionation reaction (Eq. 2) [35]:

2 $$ {2\mathrm{H}}^{+}+{{3\mathrm{NO}}_2}^{-}\to {{\mathrm{NO}}_3}^{-}+2\mathrm{NO}+{\mathrm{H}}_2\mathrm{O} $$

Hence, the decrease in peak current at low pH can be also attributed to the low stability of HNO_2_ giving rise to the creation of NO and NO_3_^−^ species. The gradual drop in the peak currents within the pH range 3.5 and 9.6 could be tentatively assigned to the decrease in the density of positively charged quaternary ammonium groups occurring at the increase of pH. At higher pH, quaternary ammonium hydroxide can be formed on the electrode surface that inhibits the electrostatic attraction of nitrite [29].

It was previously proved in many previous papers that the highest currents of nitrite oxidation were recorded in pH range between 3.0 and 4.0 [5, 13, 34–38]. In our study, the maximal peak current was observed at pH = 3.5; therefore, pH 3.5 was selected as optimum for further voltammetric determination of nitrite.

The oxidation peak potential of nitrite shifted negatively at a slope of − 89 mV/pH in the range between 1.0 and 3.5. This value is close to that expected for oxidation involving 2 e^−^ and 3 H^+^ (~ 90 mV/pH). Therefore, in an electrolyte with a low pH, nitrite can be oxidized to nitrate according to the reaction presented in Eq. 3:

3 $$ {\mathrm{H}\mathrm{NO}}_2+{\mathrm{H}}_2\mathrm{O}\to {{\mathrm{NO}}_3}^{-}+{3\mathrm{H}}^{+}+{2\mathrm{e}}^{-} $$

In electrolytes with pH between 3.5 and 9.6, one-electron and zero-proton oxidation of nitrite to nitrogen dioxide should be expected according to Eq. 4 [39]:

4 $$ {{\mathrm{NO}}_2}^{-}-{\mathrm{e}}^{-}\to {\mathrm{NO}}_2 $$

Electrode mechanism involving the formation of nitrate in strongly acidic media and formation of nitric oxide (IV) in more neutral electrolytes (at pH above pK_a_ of HNO_2_) was also observed on boron-doped diamond (BDD) electrodes [40].

**Fig. 4:**
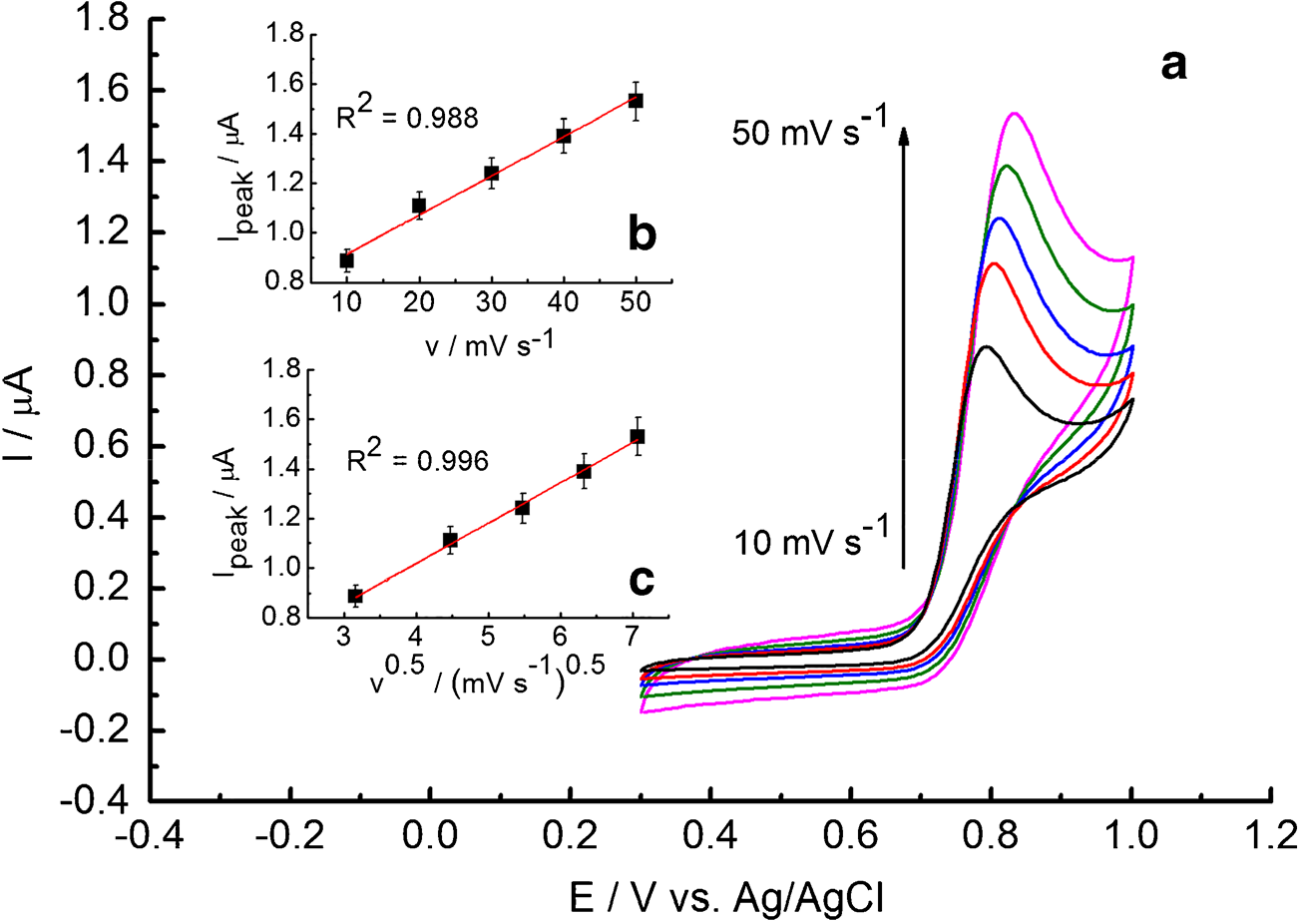
(A) CVs recorded on a GC/C_22_TMA-ASC electrode in phosphate buffer (pH 7.4) at sweep rates of 10 (a), 20 (b), 30 (c), 40 (d), and 50 mVs^−1^ (e). (B) The relationship between peak current vs. scan rate (I vs. v), *n* = 3. (C) The relationship between peak current vs. square root of scan rate (I vs. v^0.5^), *n* = 3

Cyclic voltammetry investigation with different scanning rates was performed to determine whether the NaNO_2_ oxidation is controlled by diffusion or adsorption processes on the GC/C_22_TMA-ASC electrode (Fig. 4 A). The oxidation peak current of nitrite increased with the increasing scanning rate. The linear relationship of the peak current versus scan rate was obtained from 10 mV s^−1^ to 50 mV s^−1^ with a correlation of *R*^2^ = 0.988 (Fig. 4 B). However, a better linear correlation was observed when I was plotted vs. v^0.5^ (*R*^2^ = 0.996, Fig. 4 C). Such findings indicate that nitrite oxidation is strongly influenced by both diffusion and adsorption (accumulation). Hence, it can be assumed that the mixed diffusion-adsorption process controls the oxidation at the GC/C_22_TMA-ASC electrode surface.

### Nitrite detection by DPV using GC/C_22_TMA-ASC

**Fig. 5:**
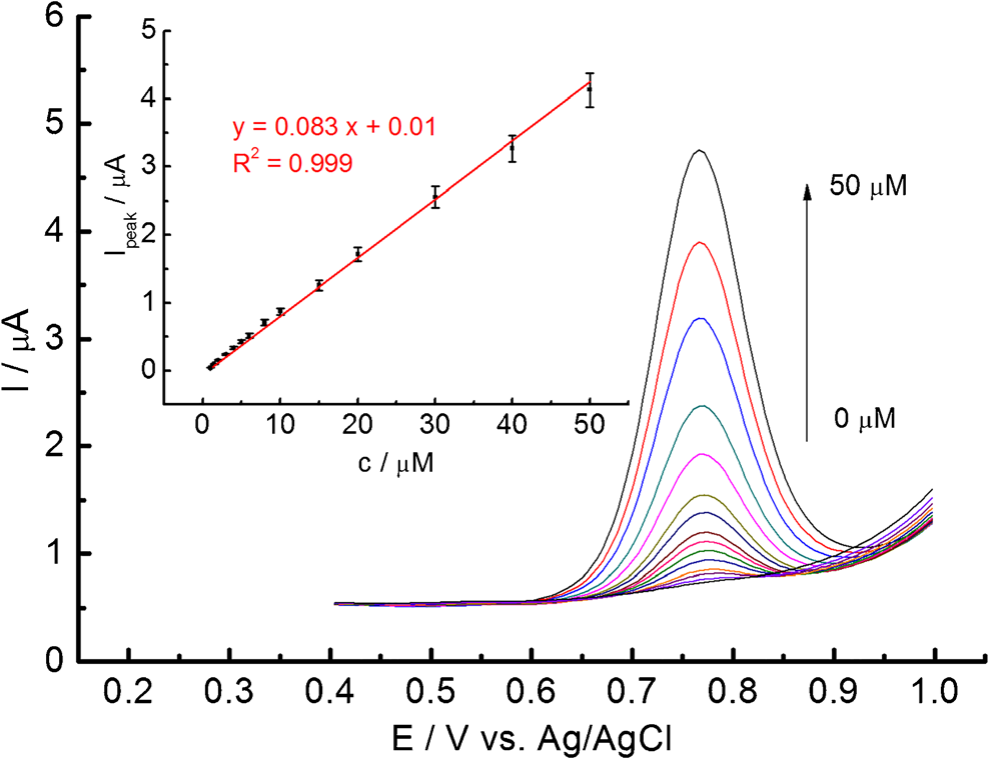
DPV curves to the increasing NaNO_2_ concentration at GC/C_22_TMA-ASC modified electrode in pH 3.5. The inset shows the corresponding I_peak_ vs. c relationship (*n* = 3)

Furthermore, DPVs of GC/C_22_TMA-ASC in the electrolyte containing different concentrations of NaNO_2_ were recorded (Fig. 5). As shown, the anodic peak current shifts to higher values by increasing NaNO_2_ concentration. Such a response enables the quantitative analysis of nitrite at GC/C_22_TMA-ASC by DPV. The plot of anodic current vs. NaNO_2_ concentration is linear in the concentration range 0.5 to 50 μM, which fits the following equation: I_peak_ (μA) = 0.024 [NaNO_2_] (μM) + 0.003 (μA), (*R*^2^ = 0.999). These results suggest that the prepared sensor possesses a high electroanalytical ability to determine nitrites.

Limits of detection (LOD) and limits of quantification (LOQ) were calculated from the plot of peak current versus concentration according to equations LOD = 3 SD/a and LOQ =10 SD/a, where SD is the standard deviation of the intercept and a is the slope of the calibration plot. LOD and LOQ were calculated to be 0.10 and 0.36 μM, respectively. The sensitivity of the GC/C_22_TMA-ASC electrode was equal to 1185 μA mM^−1^ cm^−2^ (0.083 μA μM^−1^).

### Repeatability, reproducibility, and storage stability of GC/C_22_TMA-ASC electrode

The GC/C_22_TMA-ASC electrode presented satisfactory repeatability and reproducibility for NaNO_2_ determination. Ten determinations of 10 μM NaNO_2_ were performed for a single GC/C_22_TMA-ASC (intra-day repeatability). The relative standard deviation (RSD) for 10 determinations of NaNO_2_ was 3.9%. Moreover, the series of five different GC/C_22_TMA-ASC electrodes fabricated in the same manner (inter-day reproducibility) have presented the response with an RSD of 6.59% during the voltammetric determination of 10 μM NaNO_2_.

Taking previously published reports on voltammetric sensors of nitrite into consideration (Table 2), it can be stated that GC/C_22_TMA-ASC indicates comparable and valuable analytical performance fitting within the limits of previous electrochemical sensors.

**Table 2:** An overview on recently reported nanomaterial-based electrochemical methods for the determination of nitrite

Electrode	LOD (μM)	Linear range (μM)	Sensitivity (μA mM^−1^ cm^−2^) *μA μM^−1^	Ref.
AgPs-IL-CPE^a^	3.0	50–1000	–	[41]
PEDOT-HMF^b^	0.59	50–7500	255.2	[16]
CoPcF-MWCNTs/GC^c^	0.062	0.096–340	-	[10]
GC/SWCNT/3^d^	1.08	5–200	500	[18]
PDAP-NiHCF/GCE^e^	0.0151	0.1–130	7500	[13]
Ag/HNT/MoS_2_-CPE^f^	0.70	2–425	2900	[5]
CR-GO/GCE^g^	1.00	8.9–167	-	[42]
Pd/CoPc^h^	0.1	0.2–50 and 500–5000	0.01*	[3]
Ag-rGO/GCE^i^	0.012	0.1–120	18.4	[6]
TiO_2_/CILE^j^	0.2	0.5–1500	-	[43]
POA/IL-CPE^k^	1.05	0.2–50	-	[44]
1-M-3-BIBr/CuO/SWCNTs/CPE^m^	0.5	1.0–10,000	0.0507*	[11]
AuNPs/Ti_3_C_2_T_x_-PDDA/GCE^n^	0.059	0.1–2490 and 2490–13,500	250	[12]
GC/C_22_TMA-ASC	0.1	0.5–50	1185	t.w.

^a^Silver particle-decorated carbon paste electrode based on IL

^b^Poly(3,4-ethylenodioxythiophene) hollow microflowers

^c^Glassy carbon electrode modified with tetrakis (3-trifluoromethylphenoxy) phthalocyaninato cobalt(II) on multiwalled carbon nanotubes

^d^Glassy carbon electrode modified by single-walled carbon nanotubes and 2,3,7,8,12,13,17,18-Octakis{2-[2-(3,5-dibutoxycarbonylphenoxy) ethoxy]ethylsulfanyl}porphyrazinato nickel(II)

^e^Glassy carbon electrode modified by nickel hexacyanoferrate poly(2,6-diaminopyridine) hybrid

^f^Silver/halloysite nanotube/molybdenum disulfide carbon paste electrode

^g^Chemically reduced graphene oxide modified glassy carbon electrode

^h^Glassy carbon electrode modified by cobalt phthalocyanine supported palladium nanoparticle composite

^i^Glassy carbon electrode modified by nanocomposite of reduced graphene oxide decorated with silver nanoparticle

^j^Titanium dioxide nanoparticles/IL composite electrode

^k^Poly (o-anisidine) film at the surface of IL carbon paste electrode

^m^Carbon paste electrode modified with 1-methyl-3-butylimidazolium bromide and CuO decorated single-wall carbon nanotubes

^n^Gold nanoparticles deposited on poly (dimethyl diallyl ammonium chloride)–decorated MXene (Ti_3_C_2_T_x_)

**Fig. 6:**
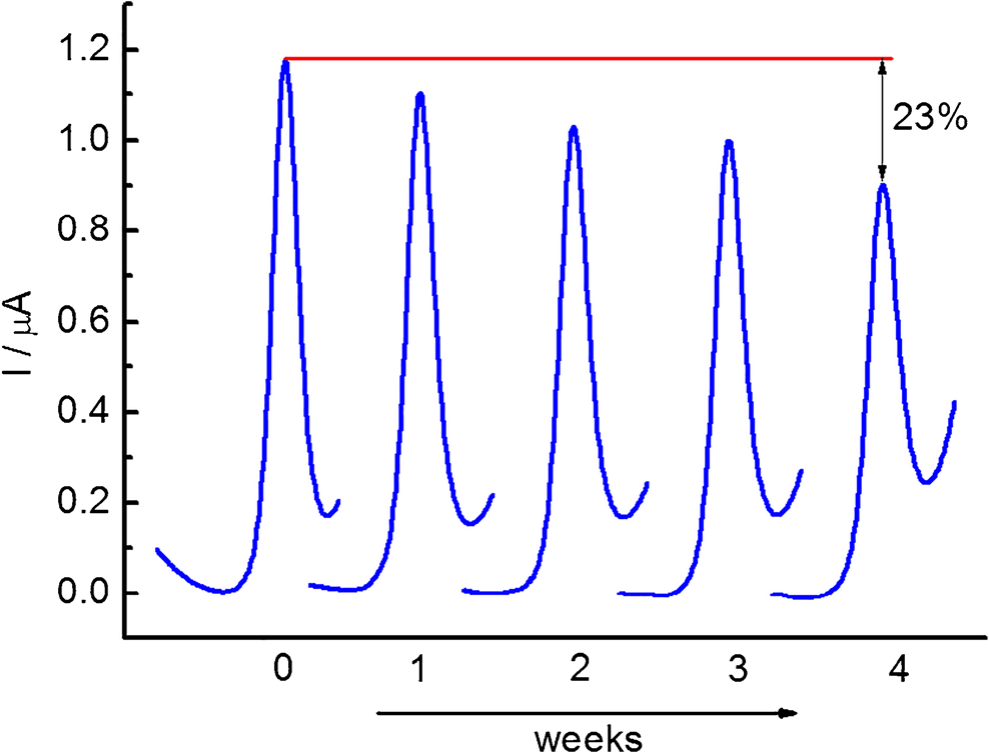
DPV curves obtained using GC/C_22_TMA-ASC in the presence of 10 μM of NaNO_2_ after 1, 2, 3, and 4 weeks storage in buffer solution (pH = 3.5)

The evaluation of fabricated sensor stability is very important for the sensor’s applicability. After the initial determination of nitrite, the GC/C_22_TMA-ASC electrode was stored in the buffer (pH = 3.5) at room temperature. The electrochemical response for the detection of nitrite was regularly tested over 4 weeks (Fig. 6). For this purpose, 3 electrodes prepared in the same manner were tested parallelly (*n* = 3). After 4 weeks, the final current decreased by 23 ± 4% of its initial value, which suggests that the electrode was reasonably stable during the test period. Good stability of the electrode can be achieved due to the hydrophobic property of the long alkyl chain that inhibits dissolution from the electrode surface.

### Interference study

**Fig. 7:**
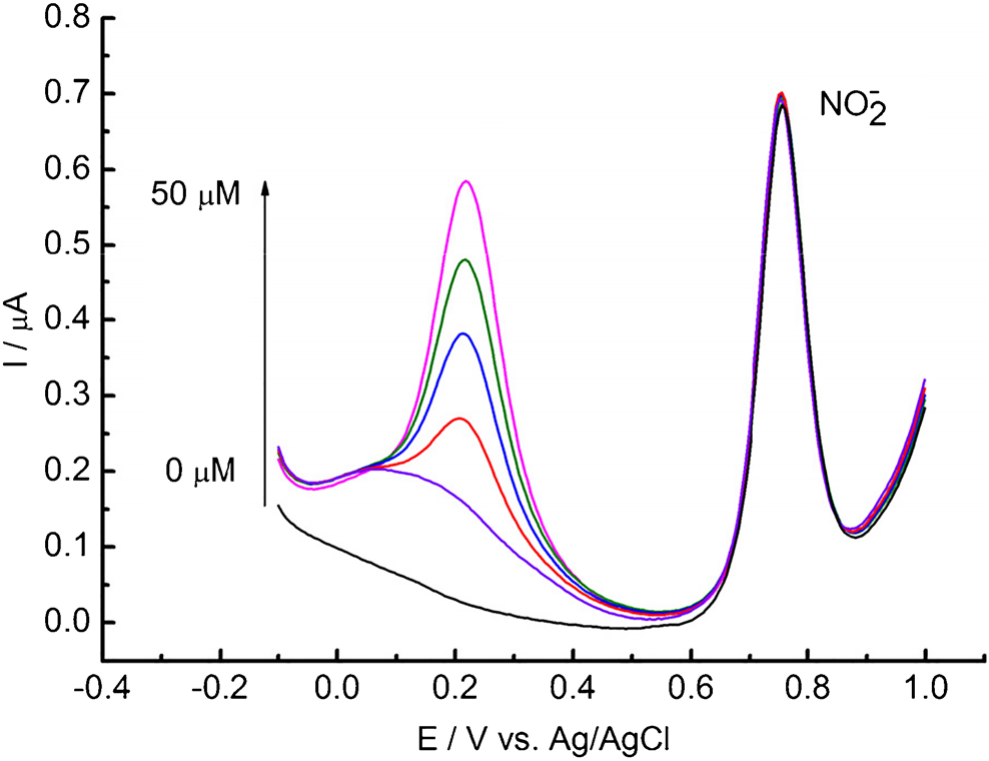
DPV curves recorded at GC/C_22_TMA-ASC of the solution containing 5 μM nitrite and the mixture of common interfering species (AA, GLU, NO_3_^−^, and Cl^−^) with various concentrations of 0, 10, 20, 30, 40, and 50 μM

The ability of selective sensing of NaNO_2_ at the GC/C_22_TMA-ASC was evaluated in the presence of a mixture of interfering substances, such as ascorbic acid (AA), glucose (GLU), nitrate, and chloride. As shown in Fig. 7, apart from a well-defined voltammetric peak of nitrite, another peak at 0.22 V can be observed with increasing concentration of interferents. This peak derives from the AA oxidation. As demonstrated, the increasing interferent concentrations produced a gradual increase in the voltammetric peak of AA; however, even the presence of a 10-fold higher concentration of AA does not interfere with the voltammetric peak from NaNO_2_ oxidation. GLU cannot be oxidized at GC/C_22_TMA-ASC in the studied potential range. Besides, no NaNO_2_ signal changes were recorded with increasing concentrations of NO_3_^−^ and Cl^−^. Additionally, the peak current of 5 μM nitrite barely changed having added 10-fold of catechol, hydroquinone, uric acid, ibuprofen, and FeCl_3_ (Fig. S4). However, the presence of 50 μM of Na_2_SO_3_ enhances the nitrite peak current by 4.7%. These results revealed that the GC/C_22_TMA-ASC sensor can be applied for the selective determination of nitrites in the presence of potential interferents.

### Determination of nitrite in commercial curing salts

To investigate the applicability of the presented voltammetric sensor in real samples, the determinations of nitrite were performed in commercially available curing salts. Nitrite is the most frequently used additive to curing salts serving as a preservative [6, 9, 17]. The samples were purchased from a local supermarket, and the content of nitrites was determined by the standard addition method.

The representative standard addition plot is shown in Fig. S5. The values of the nitrite in the studied samples are collected in Table S1. As seen, the amounts of nitrates in curing salts are in all cases very close to 0.5 wt% in all cases. The results are in good agreement with the values given by the suppliers, which indicates that the sensor can be used for the determination of nitrite in various salt samples. Recovery studies were conducted on samples by nitrite standard added (5 μM). The results imply good percent recoveries in the range from 100 to 104%, hence indicating an accurate assay.

## Conclusion

The conducted research has shown that GC/C_22_TMA-ASC electrode can be fabricated for the development of voltammetric nitrate sensors. The sensitive detection of nitrite was achieved due to the electrostatic interaction between positively charged ILs groups present on the electrode surfaces and negatively charged nitrite.

Each of the applied ILs increased the sensitivity of voltammetric nitrite determination in comparison to the unmodified GC electrode. The highest oxidation currents of nitrite were observed with the use of the electrode modified by the IL containing behenyl trimethylammonium cation and ascorbate anion (GC/C_22_TMA-ASC). The sensor demonstrated a reasonable detection limit, moderate linear concentration range, good reproducibility, high stability, and anti-interference ability. Besides, due to the simple preparation and easily renewable surface, the present approach can prove to be simple and versatile for further reference. The nitrite content in the cured salt samples tested ranges from 0.455 to 0.510% by weight.

It is essential for the proposed method to undergo further improvements in order to increase the linear range of the sensor and decrease the limit of detection. The approach presented in this paper, however, opens us up to the opportunity for further extended studies on various long alkyl chain ionic liquids for the design of high-performance electrochemical sensors. Making use of this approach for the determination of other compounds such as biomolecules, pollutants, and drugs remains a work in progress.

## Supplementary Information

ESM 1 (DOCX 627 kb)
