# Supplementary material for: Voltammetric sensor based on long alkyl chain tetraalkylammonium ionic liquids comprising ascorbate anion for determination of nitrite
Source: Mikrochim Acta. 2021 Jan 27;188(2):54. doi: 10.1007/s00604-021-04713-4 (PMC7838138; doi:10.1007/s00604-021-04713-4)
Supplement: Supplementary file 1 — (DOCX 627 kb) [file 604_2021_4713_MOESM1_ESM.docx]

**Supporting Material**

**Voltammetric sensor based on long alkyl chain tetraalkylammonium ionic liquids comprising ascorbate anion for determination of nitrite**

**Tomasz Rębiś^a*^, Michał Niemczak^b^, Patrycja Płócienniczak^a^, Juliusz Pernak^b^, Grzegorz Milczarek^a^**

*^a^Institute of Chemistry and Technical Electrochemistry, Poznan University of Technology, Berdychowo 4, 60-965 Poznan, Poland*

*^b^Department of Chemical Technology, Poznan University of Technology, ul. Berdychowo 4, Poznan 60-965, Poland*


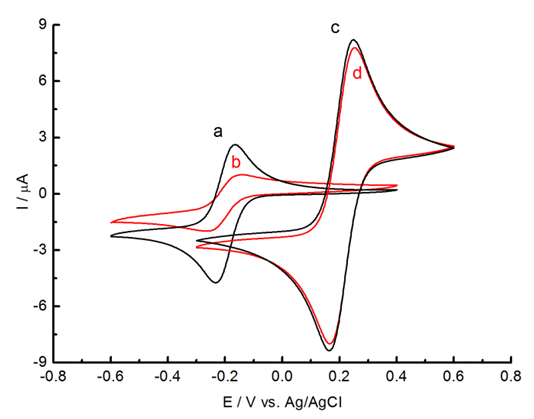


**Fig. S1.** CVs in the presence of 1 mM of Ru(NH_3_)_6_^3+^ at (a) bare GC and (b) GC/C_18_TMA-ASC and in the presence of 1 mM Fe(CN)_6_^3-/4-^ at (c) bare GC and (d) GC/C_18_TMA-ASC. Supporting electrolyte was PB solution (pH 7.4). Scan rate 10 mV s^-1^.


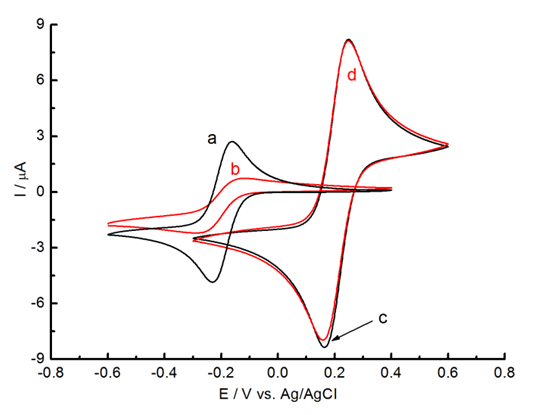


**Fig. S2.** CVs in the presence of 1 mM of Ru(NH_3_)_6_^3+^ at (a) bare GC and (b) GC/C_22_TMA-Cl and in the presence of 1 mM Fe(CN)_6_^3-/4-^ at (c) bare GC and (d) GC/C_22_TMA-Cl. Supporting electrolyte was PB solution (pH 7.4). Scan rate 10 mV s^-1^.


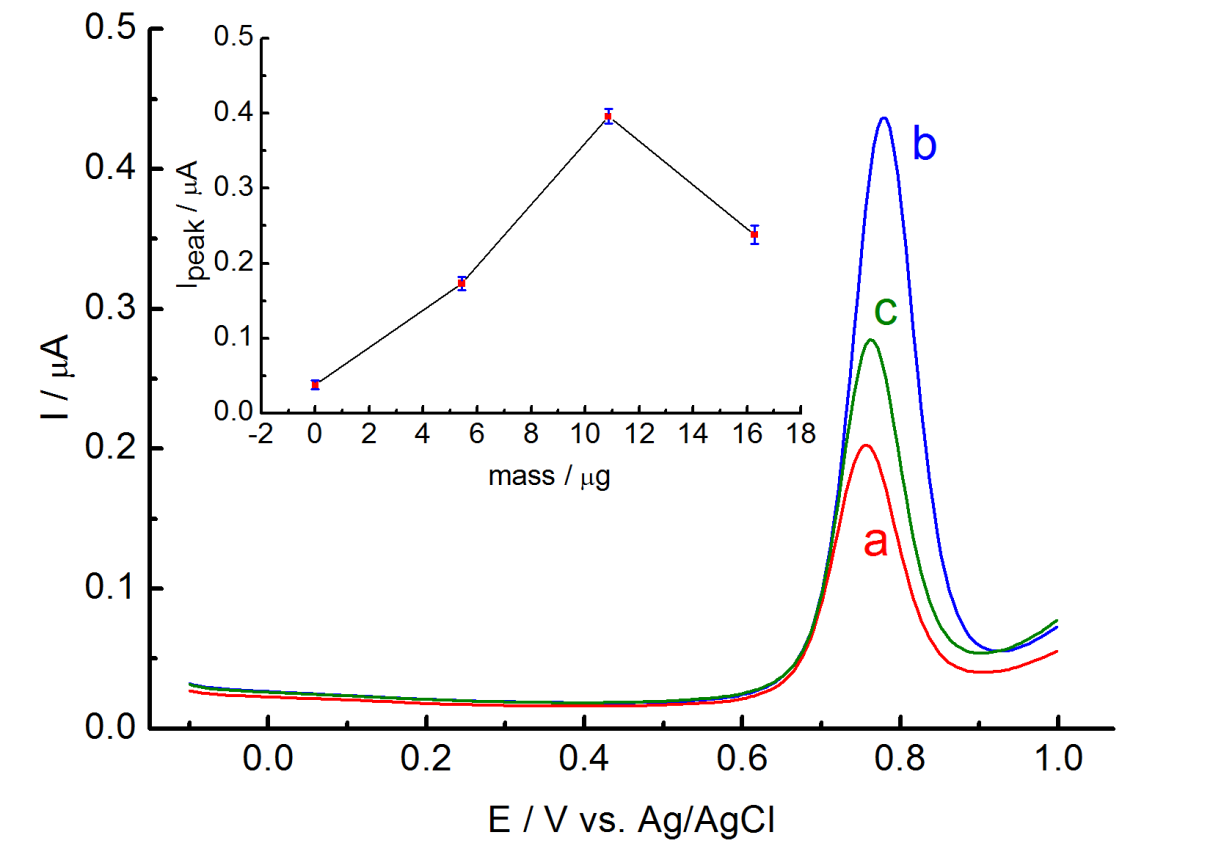


**Fig. S3.** DPV response in the presence of 5 µM NaNO_2_ for GC/C_22_TMA-ASC loaded with a different mass of C_22_TMA-ASC. Mass loading is 5.5 µg (a), 11 µg (b) and 16.5 µg (c). The inset shows the I_peak_ vs. mass relationship (n = 3).


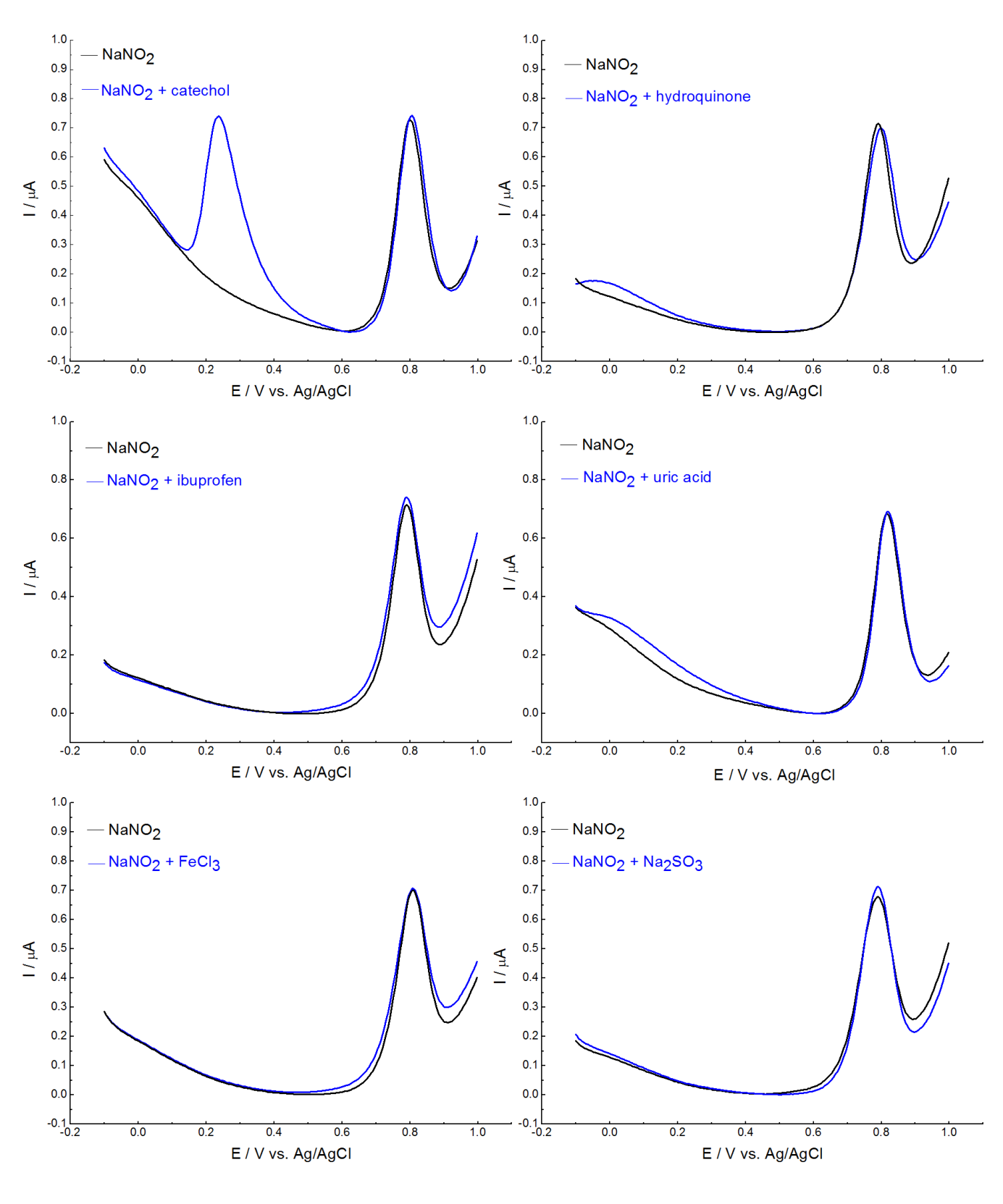
**Fig. S4.** DPV curves recorded at GC/C_22_TMA-ASC of the solution containing 5 µM nitrite and the selected interferents such as catechol, hydroquinone, ibuprofen, uric acid, FeCl_3_ and Na_2_SO_3_ with concentrations of 50 µM.


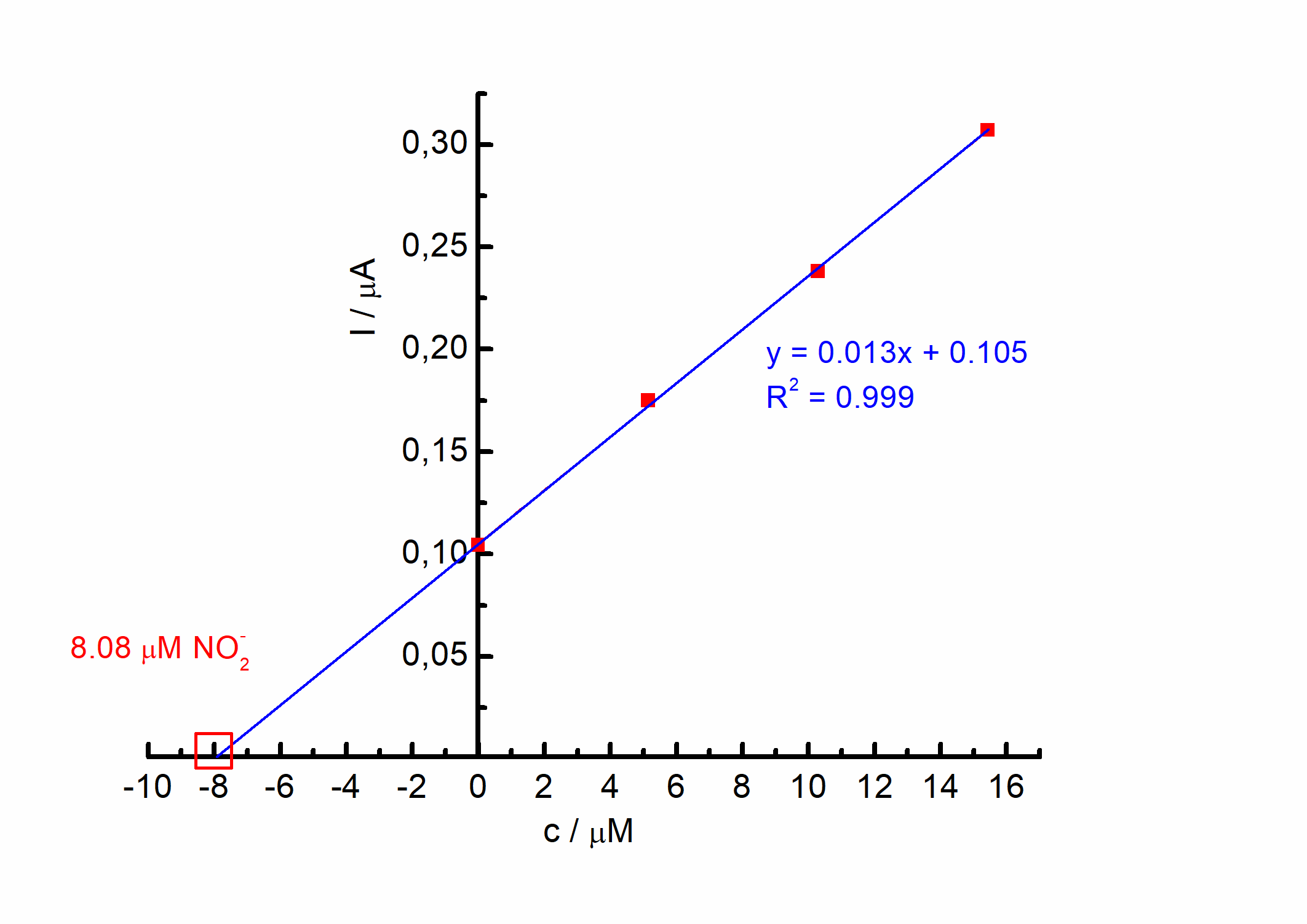


**Fig. S5.** Standard addition plot for determination of nitrite in curing salt sample at GC/C_22_TMA-ASC.

**Table S1.** Determination results of nitrite in curing salt samples using GC/C_22_TMA-ASC (n = 8)

| **Curing salt sample** | **Amount of NaNO_2_**  **µmol L^-1^**  **(mg g^-1^)** | **Added**  **µmol L^-1^** | **Found** | **Recovery**  **%** | **RSD** |
| --- | --- | --- | --- | --- | --- |
| 1 | 9.108 (5.10) | 5.0 | 14.4 | 102 | 3.8 |
| 2 | 8.201 (4.55) | 5.0 | 13.7 | 104 | 3.7 |
| 3 | 9.023 (5.04) | 5.0 | 14.0 | 100 | 4.2 |
